# Impact of respiratory infections, outdoor pollen, and socioeconomic status on associations between air pollutants and pediatric asthma hospital admissions

**DOI:** 10.1371/journal.pone.0180522

**Published:** 2017-07-18

**Authors:** Julie E. Goodman, Christine T. Loftus, Xiaobin Liu, Ke Zu

**Affiliations:** 1 Gradient, Cambridge, Massachusetts, United States of America; 2 Gradient, Seattle, Washington, United States of America; Telethon Institute for Child Health Research, AUSTRALIA

## Abstract

**Background:**

Epidemiology studies have shown that ambient concentrations of ozone and fine particulate matter (PM_2.5_) are associated with increased emergency department (ED) visits and hospital admissions (HAs) for asthma.

**Objective:**

Evaluate the impact of outdoor pollen, respiratory infections, and socioeconomic status (SES) on the associations between ambient ozone and PM_2.5_ and asthma HAs in New York City.

**Methods:**

Daily ozone, PM_2.5_, meteorological factors, pollen, and hospitalization records during 1999 to 2009 were obtained for New York City residents. Daily counts of HAs for asthma and respiratory infections were calculated for all-age and specific age groups, and for high- and low-SES communities. Generalized additive models were used to examine ambient concentrations of ozone and PM_2.5_ and asthma HAs, potential confounding effects of outdoor pollen and HAs for respiratory infections, and potential effect modification by neighborhood SES.

**Results:**

Both ozone and PM_2.5_ were statistically significantly associated with increased asthma HAs in children aged 6–18 years (per 10 ppb increase in ozone: RR = 1.0203, 95% CI: 1.0028–1.0382; per 10 μg/m^3^ increase in PM_2.5_: RR = 1.0218, 95% CI: 1.0007–1.0434), but not with total asthma HAs, or asthma HAs in other age groups. These associations were stronger for children living in the high-SES areas. Adjustment for respiratory infection HAs at various lags did not result in changes greater than 10% in the risk estimates for either ozone or PM_2.5_. In contrast, adjustment for outdoor pollen generally attenuated the estimated RRs for both ozone and PM_2.5_.

**Conclusions:**

Ambient ozone and PM_2.5_ are associated with asthma HAs in school-age children, and these associations are not modified by SES. HAs for respiratory infections do not appear to be a confounder for observed ozone- and PM_2.5_-asthma HAs associations, but pollen may be a weak confounder.

## Introduction

A number of epidemiology studies have reported positive associations between asthma exacerbation and ambient concentrations of ozone and fine particulate matter (PM_2.5_), measured as emergency department (ED) visits or hospital admissions (HAs) for asthma (*e*.*g*., [1–6]), and that children may be particularly susceptible to the effects of air pollution (*e*.*g*., see [7–10]). Many of these studies may have been vulnerable to confounding by time-varying factors such as temperature, outdoor pollen, and the start of the school year. Various methods have been developed to mitigate the effect of such confounding on effect estimates, but residual confounding remains an important source of uncertainty. Because the magnitude of associations between short-term air pollution and health effects are small, any residual confounding–even if small–could have had a substantial impact on the interpretation of study findings [11–13].

Outdoor pollen and upper respiratory infections are strong risk factors for asthma exacerbation [4, 14–19], and both exhibit short- and long-term changes over time that may correlate to some degree with temporal changes in air pollutants [16, 20]. In addition, concurrent exposures to both respiratory infections and inhaled allergens can greatly increase the risk of severe asthma exacerbations for children [21–23]. While some time series analyses of pediatric asthma and air pollution have accounted for potential confounding by pollen or upper respiratory infections with statistical adjustment for these variables (*e*.*g*., [1]), most analyses instead include a smooth function of calendar time as a surrogate measure of potential time-varying confounding. Although these confounding factors have been assessed in case-crossover designs [4, 24], to our knowledge, no time series studies have included a direct and thorough assessment of potential confounding by both factors, independently and in combination.

In addition, several recent studies reported that individual or community-level socioeconomic status (SES) modified the health effects of air pollution, and the observed effect modifications might be due to varying levels of psychosocial stress or access to healthcare resources [25–28]. We hypothesized that observed air pollution-asthma associations, as well as the potential confounding effects by respiratory infections or outdoor pollen, may vary by SES.

We conducted a time series study of asthma HAs and ambient concentrations of ozone and PM_2.5_ in New York City to determine whether results are confounded by outdoor pollen or upper respiratory infections, and whether neighborhood SES modifies the associations between air pollutants and asthma HAs with adjustment for potential confounding effects of pollen and respiratory infections.

## Methods

### Hospitalization data

We obtained hospitalization records from the New York Statewide Planning and Research Cooperative System (SPARCS) for the years 1999–2009. We identified asthma HAs by the International Statistical Classification of Diseases, 9th Revision (ICD-9) code (493), and excluded the asthma HAs that were elective admissions or had an unknown admission status. We calculated daily counts of asthma HAs for patients of all ages as well as for several age groups (under 6 years old, 6 to 18 years old, 19 to 49 years old, and 50 years old and older).

Similarly, we counted daily HAs for respiratory infections among patients of all ages as well as for children 6 to 18 years old (ICD-9: 460–466, 480–487), and daily HAs for influenza, specifically, for these age groups (ICD-9: 487). In our assessment of potential confounding by respiratory infections, we used log-transformed daily counts of HAs for respiratory infections (either all types or only influenza) as a surrogate measure of individual exposure to respiratory infections. Other epidemiology analyses of asthma have used such an ecological measure and demonstrated that it correlates strongly with short-term risk of asthma exacerbations (*e*.*g*., [20]).

Our study was approved by Chesapeake IRB (Columbia, MD).

### Environmental data

The 8-hour maximum ozone and 24-hour average PM_2.5_ concentrations were downloaded from the United States Environmental Protection Agency (US EPA) Air Quality System (AQS, https://www.epa.gov/outdoor-air-quality-data). We selected all monitors located in New York State and within a 20-mile radius of the geographic center of New York City (40.74°N, 73.91°W). The data from all selected monitors with available measurements were averaged for each day from 1999 to 2009. For sensitivity analyses, we calculated alternative daily average concentrations of pollutants by varying the location of the city center (40.73°N, 73.99°W; 40.71°N, 73.92°W) or the selection radius (15–30 miles), as well as by including nearby New Jersey and Connecticut sites.

Hourly weather data for LaGuardia Airport (Queens, NY) were obtained from the National Centers for Environmental Information (http://www.ncdc.noaa.gov/orders/qclcd/).

To estimate daily exposures to various types of outdoor aeroallergens, we relied on measurements of outdoor pollen concentrations for 2002–2006 compiled at the Fordham University's Louis Calder Biological Station in Armonk, NY, provided to us by the manager of the monitoring center. Details of the experimental technique used for sample collection and measurement of various genera of pollen are described elsewhere [29, 30]. We log-transformed daily concentrations (grains per cubic meter) for tree, weed, and total pollen, and created an indicator variable for outdoor pollen if daily concentrations exceeded the 5^th^ percentile of the concentration distribution. This dichotomized approach is based on studies of asthma morbidity and pollen exposure that show that very low concentrations of pollen are sufficient to greatly increase the risk of asthma exacerbation [17]. Because pollen data were only measured from March through October, and there were no records for some days, we imputed the missing values using the approach described by Gleason *et al*. [4]. Briefly, a day with missing pollen counts was assigned the pollen counts from the previous day for up to three consecutive days with missing values. If there were more than three consecutive days with missing pollen values, the remainder of the days were given the pollen value from the succeeding day. The log-transformed pollen concentrations from November through February were set to 0.

### Census data and stratification by SES

We stratified the counts of asthma HAs by neighborhood SES. We used patient billing US Postal Service (USPS) ZIP codes (missing for only 2.78% of records) as an approximation of ZIP Code Tabulation Areas (ZCTA), which are areal representations of USPS ZIP codes developed by the US Census Bureau for linkage to census data. We linked patient ZIP codes to census tracts using US Census Bureau 2010 databases, and then to the percentages of households in the census tract with incomes below the poverty level in the 2006–2010 five-year census. If a ZIP code matched to more than one census tract, the percentages of households in poverty for all tracts were averaged and assigned to that ZIP code. In a separate analysis, we identified each patient as living in either a relatively high or low SES ZIP code area using a cut-off of 20% of families in poverty, similar to the approach used in recent analyses of SES and asthma [28, 31].

### Statistical analysis

We assessed associations between daily counts of asthma HAs and ambient ozone or PM_2.5_ concentrations using generalized additive regression models adjusted for various potential confounders, following the approach of similar studies [3, 13]. We scaled all results to represent relative risks (RRs) of asthma HAs per 10 parts per billion (ppb) increase in ozone or 10 μg/m^3^ increase in PM_2.5_.

Our main analyses included adjustment for federal holidays, the day of the week, the beginning of the school year (September [32]), temperature, very hot and humid conditions (defined as average temperature greater than 78 degrees and relative humidity greater than 80%), and temporal trends (Eq 1). For the temporal relationship between exposure and outcome, we analyzed the average pollutant concentrations on the same day and one day prior to the day of asthma HA (*i*.*e*., lag 0–1). We calculated associations using all data between 1999 and 2009, as well as data restricted to the warm season (April through August) because ozone and PM_2.5_ concentrations are generally higher during the warm season and warm season associations are often stronger than those observed in the cold season or across the whole year (*e*.*g*., [1, 4, 9, 24, 33]).

$$\log\left(E\left(Y_{t}\right)\right)=\alpha +\beta \times Pollutant_{lag01_{t}}+\gamma \times DOW_{t}+\delta \times IHH_{t}+\eta \times Holiday_{t}+\theta \times School_{t}+ns\left(time_{t},12/year\right)+ns\left(Temp_{lag0_{t}},3\right)+ns\left(Temp_{lag12_{t}},3\right)+\upsilon_{1}\times Y_{t-1}+\upsilon_{2}\times Y_{t-2}+\upsilon_{3}\times Y_{t-3}+\upsilon_{4}\times Y_{t-4},$$ (Eq 1)

where:

*E*(*Y*_*t*_) is the expected count of asthma HAs on day *t*,
${Pollutant}_{{lag01}_{t}}$ is the ozone or PM_2.5_ average daily concentrations on day *t* and one day prior,
*DOW*_*t*_ is a categorical variable with seven levels specifying the day of the week on day *t*,
*IHH*_*t*_ is an indicator variable that equals 1 on very hot and humid days (*i*.*e*., average temperature > 80°F and relative humidity > 80%), and otherwise 0,
*Holiday*_*t*_ is an indicator variable that equals 1 if day *t* is a federal holiday, and otherwise 0,
*School*_*t*_ is an indicator variable that equals 1 if day *t* is in September, and otherwise 0,
*ns*(*time*_*t*_, 12 *df*/*year*) is a cubic spline function of time with twelve knots per year to control for temporal trends,
$ns\left({Temp_{lag0}}_{t},3\right)$ is a cubic spline function of the current-day average temperature with 3 degrees of freedom,
$ns\left({Temp_{lag12}}_{t},3\right)$ is a cubic spline function of the average temperature over the last two days with 3 degrees of freedom, and
*Y*_*t*−1_,*Y*_*t*−2_,*Y*_*t*−3_,*Y*_*t*−4_ are lag 1, lag 2, lag 3, and lag 4 day of asthma HAs counts on day *t*.

In sensitivity analyses, we evaluated whether results from our main analyses were robust against variations in model specifications, including by varying the degrees of freedom in temporal trends and varying the form of the temperature and humidity covariates (*i*.*e*., using maximum or minimum instead of average). We also tested whether the observed associations in our main analyses varied when alternative daily average concentrations of pollutants were used.

We repeated all analyses with the inclusion of residents' exposure to respiratory infections or ambient pollen, and we determined whether adjustment for these factors affected main effect estimates between air pollutants and daily asthma HAs. To assess confounding by respiratory infections, we adjusted for the HAs for total respiratory infections or for influenza among school-age children (6–18 years) at various lags (*i*.*e*., lag 0–1, lag 2–3, lag 4–6, lag 0–6). We evaluated confounding by ambient pollen by adjusting for outdoor tree, weed, or total pollen, either as a continuous variable or a binary variable, at various lags (*i*.*e*., lag 0–1, lag 2–3, lag 4–6, lag 0–6).

We also explored whether respiratory infections and pollen confound associations between air pollution and asthma HAs synergistically; that is, whether controlling for periods of both high respiratory infections and high pollen exposure affect the magnitude of associations between air pollution and asthma. To do so, we chose the ambient pollen and respiratory infection variables that have the strongest confounding effects on the main model, and repeated the main analysis for each pollutant with the inclusion of both factors, as well as their interaction term.

Finally, we evaluated whether SES modified the observed air pollution-asthma HAs associations, by including an indicator variable for patients living in relatively high- or low-SES neighborhoods and an interaction term between the indicator variable and the air pollutant in the main model. In an exploratory analysis, we also evaluated the confounding effect of outdoor pollen separately in the high- and low-SES communities.

All statistical analyses were conducted with SAS 9.4 (SAS Institute Inc., Cary, NC).

## Results

Daily counts of HAs for asthma and respiratory infections, daily ambient ozone, PM_2.5_, pollen concentrations, and daily temperatures in New York City are presented in Table 1 and S1 Table. There were a total of 295,497 (average daily count of 73.5) asthma HAs in New York City from 1999 to 2009, which included 113,144 (38.3%) patients who lived in high SES communities (mean daily counts = 28.2) and 179,743 (60.8%) who lived in low SES communities (mean daily counts = 44.7). Daily counts of respiratory infection HAs ranged from 0 to 534, with an average of 156.4. Daily pollen concentrations ranged from 0 to 16,425.2 grains/m^3^, with a mean of 234.6 grains/m^3^. Average daily concentrations of ozone and PM_2.5_ were 30.7 ppb and 13.8 μg/m^3^, respectively. Both ozone and PM_2.5_ were significantly correlated with temperatures and total weed pollens (S2 Table).

**Table 1:** Daily hospital admissions for asthma and upper respiratory infections, air pollutants, outdoor pollen, and temperature in New York City, from 1999 to 2009.

	Percentile					
	Mean (SD)	Min	25^th^	50^th^	75^th^	Max
**Hospital Admissions for Asthma (N)**						
Total	73.54 (22.39)	0	57	74	88	175
Age < 6 years	17.39 (8.39)	0	11	17	22	70
Age 6–18 years	12.02 (7.37)	0	6	11	16	60
Age 19–49 years	19.57 (6.86)	0	15	19	24	62
Age 50+ years	24.56 (24.56)	0	19	24	29	66
High SES	28.16 (9.66)	0	22	28	34	75
Low SES	44.73 (14.40)	0	34	44	54	107
**Hospital Admissions for Upper Respiratory Infections (N)**						
;Total	156.35 (48.68)	0	123	151	181	534
Age < 6 years	36.49 (21.17)	0	20	32	47	124
Age 6–18 years	7.50 (4.04)	0	5	7	10	43
Age 19–49 years	24.74 (8.43)	0	19	24	29	75
Age 50+ years	87.62 (26.30)	0	72	85	99	333
High SES	85.69 (27.23)	0	68	82	98	308
Low SES	68.09 (23.19)	0	51	66	81	217
**Air Pollutants**						
8-hour max ozone (ppb)	30.73 (16.90)	2.00	18.60	28.00	39.90	105.40
24-hour average PM_2.5_ (μg/m^3^)	13.76 (8.25)	2.13	7.67	11.66	17.70	80.70
**Outdoor Pollen**^**a**^ **(grains/m**^**3**^**)**						
Total pollen	234.55 (825.79)	0	4.42	15.80	92.80	16,425.15
Total tree pollen	223.83 (822.24)	0	0.00	4.42	73.82	16,405.35
Total weed pollen	4.81 (10.48)	0	0.00	0.00	5.40	114.90
**Temperature (°F)**						
Daily average temperature	55.78 (17.04)	8.80	42.10	55.80	71.00	93.00
Daily minimum temperature	49.67 (16.67)	2.00	37.00	50.00	65.00	86.00
Daily maximum temperature	62.18 (17.95)	16.00	48.00	62.10	78.00	101.00

PM_2.5_ = Fine Particulate Matter; ppb = Parts Per Billion; SD = Standard Deviation; SES = Socioeconomic Status; μg/m^3^ = Microgram Per Cubic Meter.

(a) Pollen data are available from March to October, 2002–2006.

Adjusted RRs for total and age-specific asthma HAs associated with ozone and PM_2.5_ concentrations at lag 0–1 day are presented in Table 2. In all-year analyses, increases in ozone and PM_2.5_ concentrations were statistically significantly associated with increased asthma HAs in children aged 6–18 years (ozone: RR = 1.0203, 95% confidence interval [CI]: 1.0028–1.0382; PM_2.5_: RR = 1.0218, 95% CI: 1.0007–1.0434) but not with total asthma HAs or asthma HAs in other age groups. The point estimates for the two pollutants and asthma HAs in school-age children appeared to be slightly stronger for warm-season analyses (ozone: RR = 1.0271, 95% CI: 1.0039–1.0508; PM_2.5_: RR = 1.0469, 95% CI: 1.0094–1.0858). We also conducted a series of sensitivity analyses with different model and covariate specifications (S3 Table). In general, the effect estimates for ozone and PM_2.5_ were robust to various specifications of temporal and meteorological variables. The results also did not change when we used alternative daily average concentrations of pollutants (S4 Table).

**Table 2:** Relative risks^a^ of hospital admissions for asthma per 10 ppb increase in ozone concentrations or per 10 μg/m^3^ increase in PM_2.5_ concentrations in New York City, from 1999 to 2009.

Hospital Admissions for Asthma	All Year				Warm Season			
	Ozone		PM_2.5_		Ozone		PM_2.5_	
	RR (95% CI)	p-value	RR (95% CI)	p-value	RR (95% CI)	p-value	RR (95% CI)	p-value
Total	1.0066	0.1	1.0053	0.3	1.0068	0.2	1.0132	0.1
	(0.9988, 1.0145)		(0.9957, 1.015)		(0.9969, 1.0167)		(0.9974, 1.0294)	
Age < 6 years	1.0001	1.0	1.0096	0.2	0.9964	0.7	1.0208	0.1
	(0.9870, 1.0133)		(0.9936, 1.0259)		(0.9795, 1.0137)		(0.993, 1.0491)	
Age 6–18 years	1.0203	0.02	1.0218	0.04	1.0271	0.02	1.0469	0.01
	(1.0028, 1.0382)		(1.0007, 1.0434)		(1.0039, 1.0508)		(1.0094, 1.0858)	
Age 19–49 years	1.0112	0.07	0.9952	0.5	1.0149	0.05	1.0156	0.2
	(0.9991, 1.0234)		(0.9805, 1.0101)		(0.9998, 1.0302)		(0.9917, 1.04)	
Age 50+ years	0.9992	0.9	1.0035	0.6	0.9987	0.9	0.9948	0.6
	(0.9881, 1.0104)		(0.9897, 1.0175)		(0.9851, 1.0125)		(0.973, 1.0171)	

CI = Confidence Interval; d.f. = Degree of Freedom; PM_2.5_ = Fine Particulate Matter; ppb = Parts Per Billion; RR = Relative Risk; μg/m^3^ = Microgram Per Cubic Meter.

(a) Relative risks were estimated from generalized additive models for lag 0–1 day air pollutants with adjustment for cubic splines of calendar time (12 d.f. per year), cubic splines of same-day temperature (3 d.f.), cubic splines of lag 1–2 day temperature (3 d.f.), start of school, very hot and humid day, day of the week, and public holidays.

**Fig 1:**
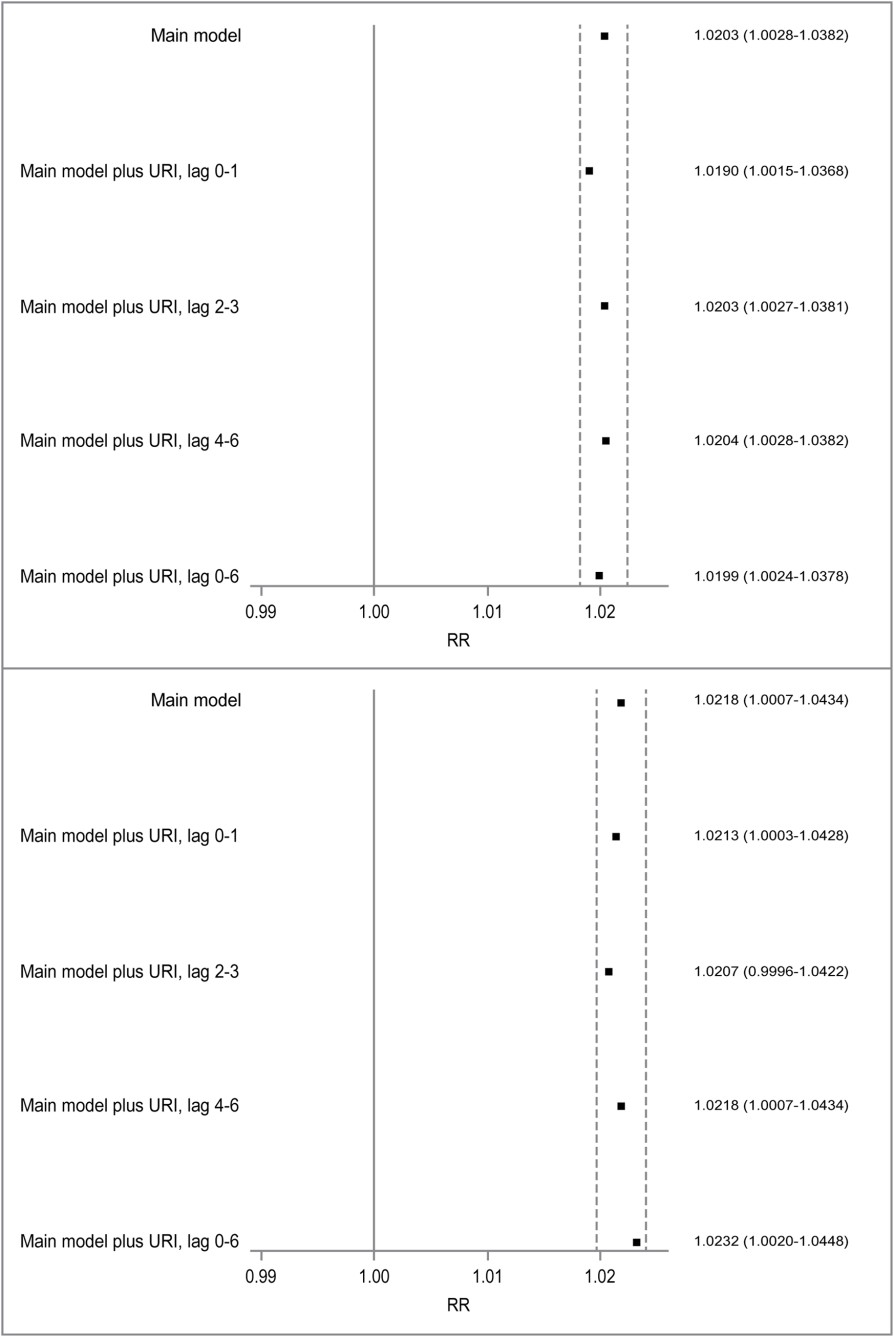
Confounding effects of hospital admissions for Upper Respiratory Infections (URI) on the associations between asthma hospital admissions in school-age children and ozone (top) and PM_2.5_ (bottom). URI is included in the main model as log-transformed daily counts of all-age URI HA. Squares are point estimates for relative risks of asthma hospital admissions associated with increases in air pollutants. The dashed lines represent ±10% changes in the point estimates from the main model.

We evaluated potential confounding of observed associations between asthma HAs in children aged 6–18 years and ozone and PM_2.5_ by respiratory infections (Fig 1). Adjustment for respiratory infection HAs at various lags resulted in minimal changes (<10%) in the risk estimates for both ozone and PM_2.5_, indicating respiratory infections did not materially confound the observed associations. Using HAs for influenza instead of all respiratory infections (S1 Fig) did not change the results in any significant way. Adjustment for respiratory infection HAs in children aged 6–18 years old instead of respiratory infections for all ages also did not change the results (S2 Fig).

**Fig 2:**
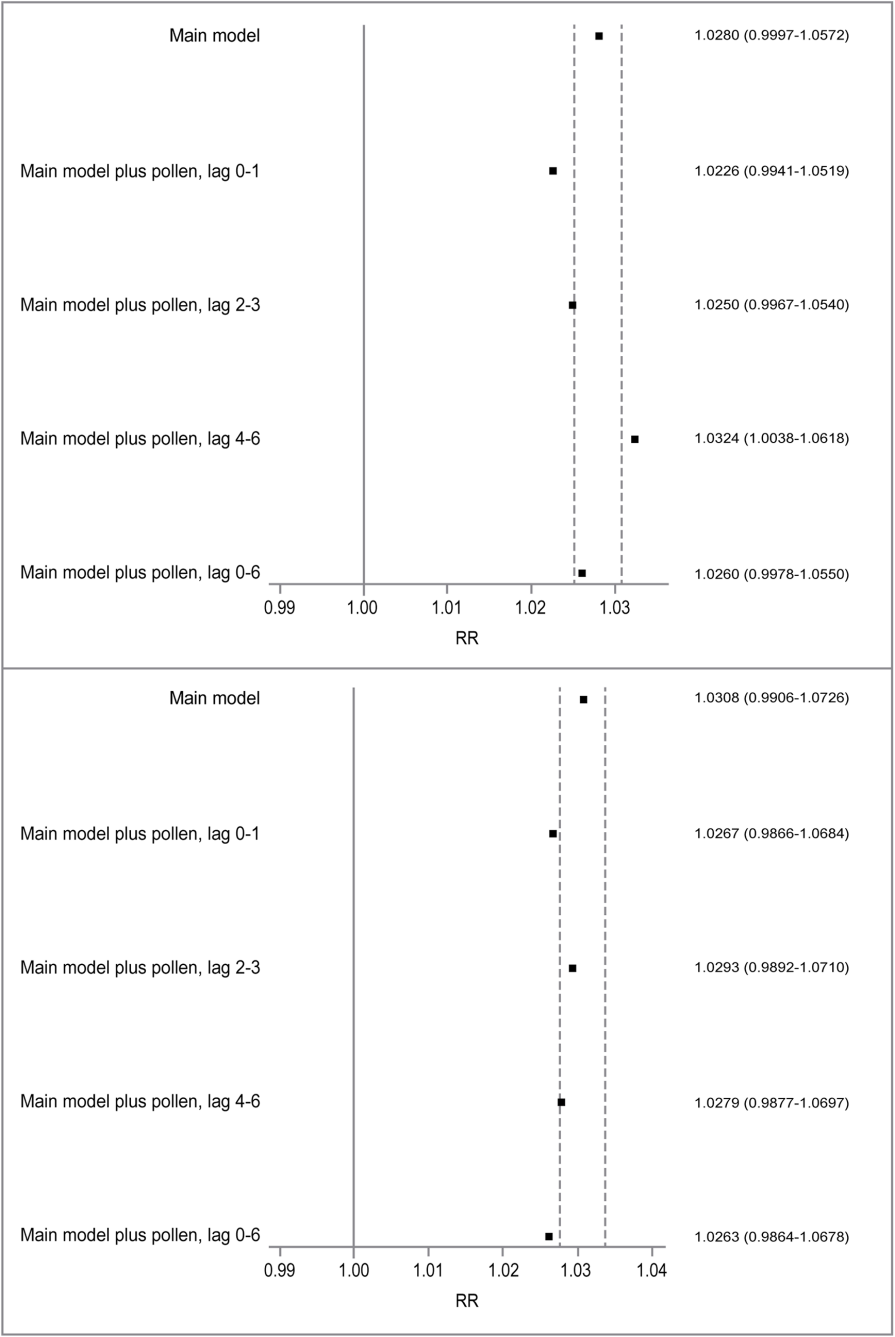
Confounding effects of outdoor pollen on the associations between asthma hospital admissions in school-age children and ozone (top) and PM_2.5_ (bottom). Squares are point estimates for relative risks of asthma hospital admissions associated with increases in air pollutants. The dashed lines represent ±10% changes in the point estimates from the main model.

We next evaluated daily pollen levels as a potential confounder for the ozone- and PM_2.5_-asthma associations in children aged 6–18 years (Fig 2). We restricted our analysis to the warm seasons of 2002–2006 (when pollen data were available), and the main models yielded non-significant results for both ozone and PM_2.5_. Inclusion of log-transformed pollen counts as a linear term generally attenuated the estimated RRs for both ozone and PM_2.5_, except for pollen counts at lag 4–6 day, which resulted in an increased RR for ozone. The results did not change with the adjustment for outdoor pollen as a binary variable (S3 Fig). Using total weed pollen or total tree pollen instead of the total pollen led to the same conclusions (S4 Fig).

We also evaluated the combined effect of respiratory infections and pollen on the results of our main analyses (S5 Fig). We only considered the lags of respiratory infections and pollen that had the largest observed confounding effect when evaluated independently (ozone: lag 0–1 for pollen, lag 0–1 for respiratory infections; PM_2.5_: lag 0–6 for pollen, lag 0–6 for respiratory infections). Inclusion of both respiratory infections and pollen, as well as their interaction term (p_interaction_ = 0.0522 and p_interaction_ = 0.0536 in the ozone and PM_2.5_ models, respectively), attenuated the estimated association between both pollutants and asthma HAs in children aged 6–18 years.

We next evaluated whether SES modifies the observed effects of ozone and PM_2.5_ on asthma HAs with adjustment for outdoor pollen. We selected pollen at lag 0–1 for the models of ozone and asthma HAs, and pollen at lag 0–6 for the models of PM_2.5_, because these were the lag times associated with the strongest confounding effects (Fig 2). As shown in Table 3, the observed associations between asthma HAs and the two pollutants, none of which were statistically significant, appeared to be similar between high- and low-SES communities.

**Table 3:** Relative risks^a^ of hospital admissions for asthma per 10 ppb increase in ozone concentrations or per 10 μg/m3 increase in PM_2.5_ concentrations at Lag 0–1 in New York City, stratified by socioeconomic status, during warm seasons from 2002 to 2006.

Asthma HAs	Warm season 02–06					
	Ozone^a^			PM_2.5_^a^		
	High-SES	Low-SES	P of SES-ozone Interaction	High-SES	Low-SES	P of SES-PM_2.5_ Interaction
All ages	1.0025	1.0034	0.8509	1.0085	1.0145	0.5408
	(0.9902, 1.0148)	(0.9920, 1.0149)		(0.9887, 1.0286)	(0.9970, 1.0323)	
Age < 6 y.o.	1.003	0.9929	0.2626	0.9979	1.0103	0.4999
	(0.9804, 1.0261)	(0.9724, 1.0139)		(0.9616, 1.0356)	(0.9786, 1.0430)	
Age 6–18 y.o.	1.0269	1.0211	0.6326	1.0212	1.034	0.5897
	(0.9965, 1.0581)	(0.9935, 1.0495)		(0.9736, 1.0711)	(0.9935, 1.0762)	
Age 19–49 y.o.	0.9989	0.9979	0.8903	0.9985	1.0148	0.3255
	(0.9789, 1.0193)	(0.9795, 1.0166)		(0.9658, 1.0324)	(0.9863, 1.0441)	
Age 50+ y.o.	1.0023	1.0053	0.6645	1.0085	1.013	0.7568
	(0.9846, 1.0204)	(0.9882, 1.0226)		(0.9797, 1.0381)	(0.9863, 1.0404)	

d.f. = Degree of Freedom; PM_2.5_ = Fine Particulate Matter; ppb = Parts Per Billion; SES = Socioeconomic Status; μg/m^3^ = Microgram Per Cubic Meter.

(a) Relative risks were estimated from generalized additive models for outdoor pollen, lag 0–1 day air pollutants with adjustment for cubic splines of calendar time (12 d.f. per year), cubic splines of same-day temperature (3 d.f.), cubic splines of lag 1–2 day temperature (3 d.f.), start of school, very hot and humid day, day of the week, and public holidays.

Finally, we explored whether the confounding effects by pollen vary by lag time, air pollutant, or SES (S6 Fig). We evaluated the changes in effect estimates of ozone and PM_2.5_ with adjustment for outdoor pollen levels at different lag times in high- and low-SES communities separately. In general, the confounding effect of pollen at various lag times appeared to be slightly stronger for ozone in high-SES communities, and for PM_2.5_ in low-SES communities. However, with the exception of ozone with adjustment for pollen at lag 4–6 in low-SES communities, all of the effect estimates for ozone and PM_2.5_ were small in magnitude and not statistically significant.

## Discussion

We conducted a time series analysis of ambient ozone and PM_2.5_ and daily counts of asthma HAs in New York City, and observed small elevations in the risk of asthma HAs among school-age children following elevated concentrations of pollutants. HAs for respiratory infections do not appear to be a confounder for observed air pollutant-asthma HAs associations, but pollen may be a weak confounder. SES did not appear to modify the observed associations between asthma HAs and air pollutants.

Our findings of increased asthma HAs associated with air pollutant exposures are consistent with several other time series studies. Recently, a meta-analysis of published epidemiology studies of air pollution and asthma HAs or ED visits reported a meta-RR of 1.008 (95% CI: 1.005–1.012; n = 42 studies) per 10 ppb increase in ozone and 1.025 (95% CI: 1.013–1.031; n = 20 studies) per 10 μg/m^3^ increase in PM_2.5_ for children [9]. Lim *et al*. [10] conducted a systematic review of PM_2.5_ exposure and reported a meta-RR of 1.048 (95% CI: 1.028–1.067) when pooling results of 26 studies of HAs and ED visits for pediatric asthma per 10 μg/m^3^ increase in PM_2.5_ exposure. Notably, both meta-analyses found a high degree of heterogeneity (>80%) among studies.

Some studies have observed evidence that respiratory infections confound associations between short-term air pollution exposure and asthma [1, 14, 20]. In contrast, we observed no compelling evidence that associations were confounded by HAs for respiratory infections in New York City. It is possible that daily counts of HAs for respiratory infections may not accurately represent the temporal patterns of residents' exposure to respiratory infections. Therefore, we cannot rule out confounding by respiratory infections in our study entirely.

Several studies have reported strong associations between outdoor pollen and other aeroallergens and asthma exacerbations [4, 17, 18, 30, 34], but only a few presented evidence that pollen may confound the associations between air pollution and asthma [4, 6]. In our analyses, pollen appeared to be a weak negative confounder for the associations between asthma HAs and ozone and PM_2.5_. However, the nature of confounding by pollen is complex due to several uncertainties. The risk of respiratory effects from pollen exposure varies significantly between individuals and is largely determined by allergic sensitization to the specific pollen genera [19]. Also, respiratory health risks can vary strongly by specific genera of pollen [4, 18]. Furthermore, there is uncertainty regarding the true shape of the dose-response relationship between pollen and respiratory effects [17] and the lag relationship between pollen exposure and health effects [4, 18]. Finally, the strength of the relationship between pollen and respiratory effects may change across the course of a pollen season, with risks declining later in the pollen season [30], which in turn could affect the confounding effect of pollen. Future analyses of pollen as a potential confounder for the associations between air pollutants and asthma should aim to account for these uncertainties.

Several researchers have hypothesized that low-resource individuals and communities are more vulnerable to the effects of air pollution [26, 28]. However, we did not observe strong evidence for interactions between air pollutants and SES in our analysis. Other published epidemiology studies have reported mixed results. For example, Wilhelm *et al*. [25] observed stronger associations between ozone and asthma among relatively high resource neighborhoods, while others found evidence of increased asthma susceptibility to air pollution in lower SES or otherwise lower resource communities [26, 27]. These mixed findings could be due to heterogeneous populations, local conditions, analytical uncertainties, such as the geographical resolution for estimates of SES (*e*.*g*., zip code *vs*. census tracts *vs*. census blocks), and whether any true effect modifications are linear. O'Lenick *et al*. [28], for example, observed a U-shaped relationship between the modification of asthma-air pollution associations by SES: associations were stronger in communities of both the lowest and highest quartiles of SES.

In an exploratory analysis, we observed slightly stronger confounding effects of pollen on the association between ozone and asthma HAs among school-age children in high-SES areas. We hypothesize that this may be because of differing access to healthcare resources and treatments for allergic airway diseases. For example, it has been shown that a child's vulnerability to respiratory effects of seasonal pollen exposure is affected by whether or not the child is taking asthma maintenance medication [17].

To our knowledge, ours is the first time series study of air pollution and asthma to examine potential confounding by pollen and respiratory infections, including an evaluation of combined effects of these two factors, and, specifically, an evaluation of whether the nature of confounding varies by neighborhood SES. Strengths of our study include the large sample size and our rigorous assessment of model specification in statistical analyses. We also used measurements of ambient pollen in New York City from a database of uniquely high-quality pollen data [30].

We also note several limitations of our analyses. As is the case for all time series air pollution analyses, our study is ecological in nature and provides limited insight into individual-level exposures and health risks. Because we relied on central site monitors to estimate population exposures to ambient air pollutants, there may be substantial exposure measurement error. The impact on health effect estimates likely depend on the amount and type of measurement errors [35, 36]. In addition, ambient pollen concentrations exhibit a high degree of geographic heterogeneity [37], thus pollen counts made at one location in New York City may not accurately represent true exposures across the study region. It is difficult to know how such spatial heterogeneity in ambient pollen concentrations impacted results.

In conclusion, we found that ambient ozone and PM_2.5_ are associated with asthma HAs among school-age children in New York City, and these associations did not vary by SES. HAs for respiratory infections do not appear to be a confounder for observed ozone- and PM_2.5_-asthma HAs associations, but there is some evidence that pollen may be a weak confounder. Future studies should consider confounding by pollen when evaluating air pollution and respiratory morbidity.

## Supporting information

S1 Table Hospital admissions for asthma and upper respiratory infections in New York City, 1999–2009. (DOCX)

S2 Table Correlations between daily air pollutants, pollen^a^ and temperatures in New York City, 1999–2009. (DOCX)

S3 Table Sensitivity analyses of asthma hospital admission and ambient air pollutants with various specifications of covariates. (DOCX)

S4 Table Sensitivity analyses of asthma hospital admission and ambient air pollutants with various lags of air pollutants. (DOCX)

S1 Fig Confounding effects of hospital admissions for influenza on the associations between asthma hospital admissions in school-age children and ozone (top) and PM_2.5_ (bottom). Squares are point estimates for relative risks of asthma hospital admissions associated with increases in air pollutants. The dashed lines represent ±10% changes in the point estimates from the main model.
(DOCX)

S2 Fig Confounding effects of hospital admissions for upper respiratory infections (uri) on the associations between asthma hospital admissions in school-age children and ozone (top) and PM_2.5_ (bottom). URI is adjusted by adding log-transformed daily URI counts for school-aged children into the main model. Squares are point estimates for relative risks of asthma hospital admissions associated with increases in air pollutants. The dashed lines represent ±10% changes in the point estimates from the main model.
(DOCX)

S3 Fig Confounding effects of outdoor pollen on the associations between asthma hospital admissions in school-age children and ozone (top) and PM_2.5_ (bottom). Squares are point estimates for relative risks of asthma hospital admissions associated with increases in air pollutants. The dashed lines represent ±10% changes in the point estimates from the main model. Outdoor pollen is defined as daily counts of > 5^th^ percentile.
(DOCX)

S4 Fig Confounding effects of outdoor tree pollen (left) and weed pollen (right) on the associations between asthma hospital admissions in school-age children and ozone (top) and PM_2.5_ (bottom). Squares are point estimates for relative risks of asthma hospital admissions associated with increases in air pollutants. The dashed lines represent ±10% changes in the point estimates from the main model.
(DOCX)

S5 Fig Combined confounding effects of hospital admissions for upper respiratory infections (URI) and outdoor pollen on the associations between asthma hospital admissions in school-age children and ozone (top) and PM_2.5_ (bottom). Squares are point estimates for relative risks of asthma hospital admissions associated with increases in air pollutants. The dashed lines represent ±10% changes in the point estimates from the main model.
(DOCX)

S6 Fig Confounding effects of outdoor pollen on the associations between asthma hospital admissions in school-age children and ozone (left) and PM_2.5_ (right), in high-SES (top) and low-SES (bottom) areas. Squares are point estimates for relative risks of asthma hospital admissions associated with increases in air pollutants. The dashed lines represent ±10% changes in the point estimates from the main model.
(DOCX)
