# Supplementary material for: Impact of respiratory infections, outdoor pollen, and socioeconomic status on associations between air pollutants and pediatric asthma hospital admissions
Source: PLoS One. 2017 Jul 18;12(7):e0180522. doi: 10.1371/journal.pone.0180522 (PMC5515410; doi:10.1371/journal.pone.0180522)
Supplement: S1 Table — (DOCX) [file pone.0180522.s001.docx]

**S1 Table. Hospital Admissions for Asthma and Upper Respiratory Infections in New York City, 1999-2009**

| **Hospital Admissions (N)** | **Total** | **High-SES Areas** | **Low-SES Areas** |
| --- | --- | --- | --- |
| **Asthma** | | | |
| All ages | 295,497 | 113,144 | 179,743 |
| Age < 6 years | 69,853 | 25,359 | 43,847 |
| Age 6-18 years | 48,316 | 16,748 | 31,267 |
| Age 19-49 years | 78,632 | 28,780 | 48,961 |
| Age 50+ years | 98,696 | 42,257 | 55,668 |
| **Upper Respiratory Infections** | | | |
| All ages | 628,222 | 344,295 | 273,570 |
| Age < 6 years | 146,634 | 60,600 | 84,336 |
| Age 6-18 years | 30,142 | 13,852 | 15,830 |
| Age 19-49 years | 99,401 | 45,743 | 51,393 |
| Age 50+ years | 352,045 | 224,100 | 122,011 |

Note:

SES = Socioeconomic Status.
