# Supplementary material for: Impact of respiratory infections, outdoor pollen, and socioeconomic status on associations between air pollutants and pediatric asthma hospital admissions
Source: PLoS One. 2017 Jul 18;12(7):e0180522. doi: 10.1371/journal.pone.0180522 (PMC5515410; doi:10.1371/journal.pone.0180522)
Supplement: S2 Table — (DOCX) [file pone.0180522.s002.docx]

**S2 Table. Correlations Between Daily Air Pollutants, Pollen^a^ and Temperatures in New York City, 1999-2009**

| **Correlation Coefficient**  **p-value** | **8-hour Max Ozone** | **24-hour Average PM_2.5_** | **Total Pollen** | **Total Tree  Pollen** | **Total Weed Pollen** | **Average Temperature** | **Minimum Temperature** | **Maximum Temperature** |
| --- | --- | --- | --- | --- | --- | --- | --- | --- |
| 8-hour Max Ozone | 1.0 | 0.2 | 0.03 | 0.03 | 0.2 | 0.7 | 0.6 | 0.7 |
|  |  | <.0001 | 0.3 | 0.4 | <.0001 | <.0001 | <.0001 | <.0001 |
| 24-hour Average PM_2.5_ | 0.2 | 1.0 | -0.03 | -0.03 | 0.1 | 0.2 | 0.2 | 0.2 |
|  | <.0001 |  | 0.3 | 0.3 | 0.02 | <.0001 | <.0001 | <.0001 |
| Total Pollen | 0.03 | -0.03 | 1.0 | 1.0 | 0.1 | -0.1 | -0.1 | -0.03 |
|  | 0.3 | 0.3 |  | <.0001 | 0.005 | 0.01 | 0.0003 | 0.3 |
| Total Tree Pollen | 0.03 | -0.03 | 1.0 | 1.0 | 0.1 | -0.1 | -0.1 | -0.03 |
|  | 0.4 | 0.3 | <.0001 |  | 0.02 | 0.01 | 0.0001 | 0.2 |
| Total Weed Pollen | 0.2 | 0.1 | 0.1 | 0.1 | 1.0 | 0.3 | 0.3 | 0.3 |
|  | <.0001 | 0.02 | 0.01 | 0.02 |  | <.0001 | <.0001 | <.0001 |
| Average Temperature | 0.7 | 0.2 | -0.1 | -0.1 | 0.3 | 1.0 | 1.0 | 1.0 |
|  | <.0001 | <.0001 | 0.01 | 0.01 | <.0001 |  | <.0001 | <.0001 |
| Minimum Temperature | 0.6 | 0.2 | -0.1 | -0.1 | 0.3 | 1.0 | 1.0 | 1.0 |
|  | <.0001 | <.0001 | 0.0003 | 0.0001 | <.0001 | <.0001 |  | <.0001 |
| Maximum Temperature | 0.7 | 0.2 | -0.03 | -0.03 | 0.3 | 1.0 | 1.0 | 1.0 |
|  | <.0001 | <.0001 | 0.3 | 0.2 | <.0001 | <.0001 | <.0001 |  |

Notes:

PM_2.5_ = Fine Particulate Matter.

(a) Pollen data are only available for March-October, 2002-2006.
