# Supplementary material for: Impact of respiratory infections, outdoor pollen, and socioeconomic status on associations between air pollutants and pediatric asthma hospital admissions
Source: PLoS One. 2017 Jul 18;12(7):e0180522. doi: 10.1371/journal.pone.0180522 (PMC5515410; doi:10.1371/journal.pone.0180522)
Supplement: S3 Table — (DOCX) [file pone.0180522.s003.docx]

**S3 Table. Sensitivity Analyses of Asthma Hospital Admission and Ambient Air Pollutants with Various Specifications of Covariates**

| **Hospital Admissions for Asthma** | **Ozone^a^** | **PM_2.5_^b^** |
| --- | --- | --- |
|  | **RR (95% CI)** | **RR (95% CI)** |
| **Specification of Temporal Splines** | | |
| ***All ages*** | | |
| 12 d.f. per year (main model^c^) | 1.0066  (0.9988-1.0145) | 1.0053  (0.9957-1.0150) |
| 8 d.f. per year | 1.0064  (0.9986-1.0143) | 1.0067  (0.9971-1.0164) |
| 16 d.f. per year | 1.008  (1.0002-1.0158) | 1.0021  (0.9925-1.0118) |
| ***Age 6-18 years*** | | |
| 12 d.f. per year (main model^c^) | 1.0203  (1.0028-1.0382) | 1.0218  (1.0007-1.0434) |
| 8 d.f. per year | 1.0189  (1.0015-1.0367) | 1.0242  (1.0031-1.0458) |
| 16 d.f. per year | 1.0268  (1.0091-1.0447) | 1.0175  (0.9964-1.0390) |
| **Specification of Meteorological Variables** | | |
| ***All ages*** | | |
| Main model^c^ | 1.0066  (0.9988-1.0145) | 1.0053  (0.9957-1.0150) |
| Model 1^d^ | 1.0066  (0.9986-1.0147) | 1.006  (0.9965-1.0156) |
| Model 2^e^ | 1.0074  (1.0004-1.0145) | 1.0064  (0.9971-1.0159) |
| Model 3^f^ | 1.0063  (0.9978-1.0149) | 1.0051  (0.9950-1.0154) |
| ***Age 6-18 years*** | | |
| Main model^c^ | 1.0203  (1.0028-1.0382) | 1.0218  (1.0007-1.0434) |
| Model 1^d^ | 1.0167  (0.9987-1.0349) | 1.0201  (0.9992-1.0414) |
| Model 2^e^ | 1.0193  (1.0034-1.0355) | 1.0239  (1.0032-1.0450) |
| Model 3^f^ | 1.0131  (0.9941-1.0326) | 1.0224  (1.0002-1.0451) |

Notes:

CI = Confidence Interval; d. f. = Degree of Freedom ; PM_2.5_ = Fine Particulate Matter; ppb = Parts Per Billion; RR = Relative Risk; μg/m^3^ = Microgram Per Cubic Meter.

(a) RRs and 95% CIs were associated with a 10 ppb increase in lag 0-1 day ozone concentrations.

(b) RRs and 95% CIs were associated with a 10 μg/m^3^ increase in lag 0-1 day PM_2.5_ concentrations.

(c) Main model is the generalized additive model adjusted for cubic splines of calendar time (12 d.f. per year), cubic splines of same-day average temperature (3 d.f.), cubic splines of lag 1-2 day temperature (3 d.f.), start of school, very hot and humid day, day of the week, and federal holidays.

(d) Model 1 replaces daily average temperature with daily maximum temperature in the main model.

(e) Model 2 replaces daily average temperature with daily minimum temperature in the main model.

(f) Model 3 replaces the indicator for very hot and humid day with cubic splines of same-day average relative humidity (3 d.f.) and cubic splines of the average of lag 1-2 relative humidity (3 d.f.).
