# Supplementary material for: Impact of respiratory infections, outdoor pollen, and socioeconomic status on associations between air pollutants and pediatric asthma hospital admissions
Source: PLoS One. 2017 Jul 18;12(7):e0180522. doi: 10.1371/journal.pone.0180522 (PMC5515410; doi:10.1371/journal.pone.0180522)
Supplement: S4 Table — (DOCX) [file pone.0180522.s004.docx]

**S4 Table. Sensitivity Analyses of Asthma Hospital Admission and Ambient Air Pollutants with Various Lags of Air Pollutants**

| **Lag** | **Ozone^a^** | **PM_2.5_^b^** |
| --- | --- | --- |
|  | **RR (95% CI)** | **RR (95% CI)** |
| ***All ages*** | | |
| Lag 0-1 (main model^c^) | 1.0066  (0.9988, 1.0145) | 1.0053  (0.9957, 1.0150) |
| Lag 0 | 0.999  (0.9926, 1.0054) | 1.0046  (0.9962, 1.0130) |
| Lag 1 | 1.0087  (1.0027, 1.0147) | 1.0018  (0.9938, 1.0098) |
| Lag 2 | 1.0082  (1.0022, 1.0144) | 1.0043  (0.9961, 1.0125) |
| Lag 3 | 1.0011  (0.9958, 1.0065) | 1.0089  (1.0015, 1.0163) |
| ***Age 6-18 years*** | | |
| Lag 0-1 (main model^c^) | 1.0203  (1.0028, 1.0382) | 1.0218  (1.0007, 1.0434) |
| Lag 0 | 1.0171  (1.0028, 1.0317) | 1.0129  (0.9947, 1.0314) |
| Lag 1 | 1.0087  (0.9954, 1.0223) | 1.0185  (1.0009, 1.0363) |
| Lag 2 | 1.0088  (0.9954, 1.0225) | 1.0034  (0.9857, 1.0214) |
| Lag 3 | 1.0014  (0.9895, 1.0134) | 1.0043  (0.9884, 1.0205) |

Notes:

CI = Confidence Interval; PM_2.5_ = Fine Particulate Matter; ppb = Parts Per Billion; RR = Relative Risk; μg/m^3^ = Microgram Per Cubic Meter.

(a) RRs and 95% CIs were associated with a 10 ppb increase in lag 0-1 day ozone concentrations.

(b) RRs and 95% CIs were associated with a 10 μg/m^3^ increase in lag 0-1 day PM_2.5_ concentrations.

(c) The main model is the Poisson generalized addivtive model adjusted for cubic splines of calendar time (12 d.f. per year), cubic splines of same-day average temperature (3 d.f.), cubic splines of the average of lag 1 through 2 day temperature (3 d.f.), start of school, very hot and humid day, day of the week, and public holidays.
