# Supplementary material for: Impact of respiratory infections, outdoor pollen, and socioeconomic status on associations between air pollutants and pediatric asthma hospital admissions
Source: PLoS One. 2017 Jul 18;12(7):e0180522. doi: 10.1371/journal.pone.0180522 (PMC5515410; doi:10.1371/journal.pone.0180522)
Supplement: S1 Fig — Squares are point estimates for relative risks of asthma hospital admissions associated with increases in air pollutants. The dashed lines represent ±10% changes in the point estimates from the main model. (DOCX) [file pone.0180522.s005.docx]

**S1 Fig. Confounding Effects of Hospital Admissions for Influenza on the Associations Between Asthma Hospital Admissions in School-age Children and Ozone (top) and PM_2.5_ (bottom).** Squares are point estimates for relative risks of asthma hospital admissions associated with increases in air pollutants. The dashed lines represent ±10% changes in the point estimates from the main model.
